# Assessment of hydrogen peroxide as a bioindicator of stress in seaweed aquaculture

**DOI:** 10.1038/s41598-024-52182-5

**Published:** 2024-01-23

**Authors:** Lina Taenzer, Gunilla Toth, Colleen M. Hansel

**Affiliations:** 1 Department of Marine Chemistry and Geochemistry, Woods Hole Oceanographic Institution, Woods Hole, MA 02543 USA; 2 Department of Earth, Atmospheric and Planetary Science, Massachusetts Institute of Technology, Cambridge, MA 02139 USA; 3 Department of Marine Sciences, University of Gothenburg, Strömstad, Sweden

**Keywords:** Biogeochemistry, Marine chemistry

## Abstract

The rapid expansion in commercial seaweed farming has highlighted the need for more effective monitoring methods, and health diagnostics. The production of the reactive oxygen species (ROS) hydrogen peroxide (H_2_O_2_) is a trait that is tied to all major macroalgal groups and holds significance both for its involvement in the oxidative stress response and in the production of climatically relevant gases such as halocarbons. Observations of increased production of H_2_O_2_ by plants as a stress response, along with its comparative stability and ease of quantification in seawater in comparison to other ROS, suggest that H_2_O_2_ could be used as an indicator of health. In this study we characterized aqueous H_2_O_2_ dynamics across a diel cycle, in response to small shifts in light and temperature, as well as when exposed to acute stress. Our results reveal that exposure to acute stressors leads to rapid and sustained concentrations of H_2_O_2_ that are orders of magnitude higher than changes in H_2_O_2_ concentrations observed throughout the day. These findings provide tantalizing evidence that monitoring H_2_O_2_ could be used as a health indicator in seaweed aquaculture and serve as an early warning sign of stress.

## Introduction

By 2050, the United Nations projects that the human population will reach 9.7 billion people^1^ generating an unprecedented demand for food and clean energy. The discovery and development of new farming practices, and food resources, lie at the crux of sustainably managing this challenge. Seaweed aquaculture could provide a multi-faceted solution to the problems of hunger, nutrient deficiency, and alternative energy supplies^2–5^. The global seaweed aquaculture industry has rapidly expanded in the last decades at a growth rate of 6.2% yr^−1^ (long-term average 2000–2018), demonstrating promise as part of a sustainable solution to impending challenges^2^. However, recent years have also witnessed an increased occurrence of crop loss. For instance, in the Philippines a 15% decline in biomass of *Kappaphycus alvarezii* due to ice-ice disease alone amounted to a loss of around 310 million US dollars between 2011 and 2013^6^.

Seaweeds grown on aquaculture farms are subject to a variety of possible environmental stressors, including changes in seawater levels, temperature, salinity, metals, and nutrients^7,8^. Apart from environmental stressors, intensive macroalgal farming has been accompanied with more frequent disease outbreaks, and grazing from copepods and amphipods^9,10^. In fact, changes in abiotic factors such as rising ocean temperatures may be linked to an increased risk of grazing and incidence of disease. For example, grazing by a marine isopod (*Cymodocea japonica)* on seaweed *(Undaria pinnatifida)* is linked to an increase in ocean temperature of 3°C^11^. Although the increased prevalence of environmental stressors and disease outbreak have led to considerable economic losses, very little is known about successful mitigation strategies^12^. Current methods tend to be time- and labor-intensive such as washing algal blades in acid solution, and visually monitoring stocks for epiphytes that are then removed by hand^13^.

Reactive oxygen species (ROS) are a group of short-lived oxygen-containing molecules, that are formed by all aerobic organisms^14^. In plants, the ROS hydrogen peroxide (H_2_O_2_) is formed at various locations, including cell membranes, chloroplasts, mitochondria, and peroxisomes, often as a decay product of the ROS superoxide^15^. The accumulation of H_2_O_2_ is hindered by a variety of enzymatic and non-enzymatic antioxidant mechanisms. However, when exposed to abiotic or biotic stressors, the production of ROS outpaces scavenging, leading to a swift increase in ROS concentrations^16–18^. The association between stress and increased concentrations of ROS have led to previous applications of ROS as biomarkers of human health^19,20^, and as stress indicators in plant studies^21–25^.

Previous work has established the production of H_2_O_2_^26–28^, and presence of ROS-scavenging enzymes^29^ in various macroalgae. An increase in ROS production is a common response to environmental stressors such as grazing/wounding, desiccation, and temperature shifts. For instance, rapid releases of H_2_O_2_ have been observed in the red alga *G. conferta* when exposed to fragments of cell wall (oligoagars) or peptides from bacteria^30,31^, and in the brown alga *Laminaria digita* when exposed to alginates (which may be grazer or pathogen produced)^22^. Such oxidative bursts (encompassing a rapid release of H_2_O_2_) are thought to be controlled by light^32,33^ and serve as defense mechanisms against pathogens, prey, and surface colonization^34–36^. Increases in temperature may lead to more sustained elevated levels of ROS. For instance, in the red alga, *Kappacphycus alvarezii*, ROS production was noted to increase with temperature up to an experimental temperature of 40 °C^37^. The magnitude of the ROS response of seaweeds to desiccation may be influenced by their adaptation to particular habitats. For example, one study comparing H_2_O_2_ release by subtidal and intertidal *Ulva lactuca* after 3 h of desiccation found notably higher levels of H_2_O_2_ released by the subtidal species^21^.

In contrast to other evanescent ROS (e.g., superoxide, hydroxyl radical), H_2_O_2_ has a sufficiently long lifetime allowing it to diffuse out and away from cells. This affords it the potential as a target for monitoring in the aqueous environment. While numerous studies have now demonstrated that seaweeds produce elevated concentrations of H_2_O_2_ under certain stressors, many of these previous experiments studied intracellular concentrations, spanned just a few hours, used non-environmentally relevant conditions, or focused on segments rather than entire individuals. Thus, we lack a comprehensive understanding of how seaweeds control aqueous H_2_O_2_ dynamics in their surroundings and whether measurements of H_2_O_2_ can inform us about seaweed health.

Bromoform (CHBr_3_), is a halocarbon with ozone-depleting potential^38,39^, that is produced by macroalgae as part of an antioxidant response. The increased generation of CHBr_3_ during exposure to environmental stressors (e.g. nutrient depletion, and desiccation) has been observed in several studies^40,41^. Once released, halocarbons may help protect macroalgae from grazing and bacterial infection^24,42^. Macroalgal production of CHBr_3_ is thought to occur via the oxidation of bromide by bromoperoxidase (BrPO) with H_2_O_2_ which generates a hypohalide that subsequently reacts with either DOM in the surrounding seawater, or ketones in the seaweed to produce bromoform^43^. A literature comparison of temperate, tropical, and polar macroalgae reveals a large range in bromoform production spanning from 0 to 6000 pmol g FW^−1^ h^−1^ for CHBr_3_, with chlorophytes as the largest producers (up to 6000 pmol g FW^−1^ h^−1^), followed by rhodophytes (up to 5000 pmol g FW^−1^ h^−1^), and then phaeophytes (up to 3000 pmol g FW^−1^ h^−1^)^44^. Although production of CHBr_3_ is well established across groups of macroalgae, a reaction for which H_2_O_2_ is requisite, a direct quantitative tie to natural biological H_2_O_2_ is still missing. Yet, contemporaneous analyses of H_2_O_2_ and CHBr_3_ production by various macroalgal species could lead to a better understanding of the drivers behind the large ranges in observed production in culture studies and help constrain spatiotemporal trends in the fluxes of CHBr_3_ from the oceans to the atmosphere.

Determining whether H_2_O_2_ and CHBr_3_ can indicate physiological stress in a manner that is distinguishable from fluctuations observed under ambient, non-stressed conditions will be essential to evaluating the potential of these compounds for early detection of stress in seaweed aquaculture. In this study we characterized the aqueous H_2_O_2_ and CHBr_3_ dynamics associated with two temperate seaweeds, the chlorophyte *Ulva fenestrata* and the rhodophyte *Palmaria palmata* with the aim of determining whether aqueous H_2_O_2_ measurements could be used to differentiate stress from baseline conditions. *U. fenestrata* and *P. palmata* are promising candidates for seaweed cultivation, and thus ideal model organisms for this type of study^45–47^. Specifically, we examined (1) temporal dynamics in H_2_O_2_, (2) responses to temperature, desiccation, and grazing, and (3) CHBr_3_ production, with the goal of examining the use of H_2_O_2_ as a diagnostic management tool and the merit of monitoring natural H_2_O_2_ and CHBr_3_ production in concert.

## Results

### H_2_O_2_ temporal dynamics

**Figure 1:**
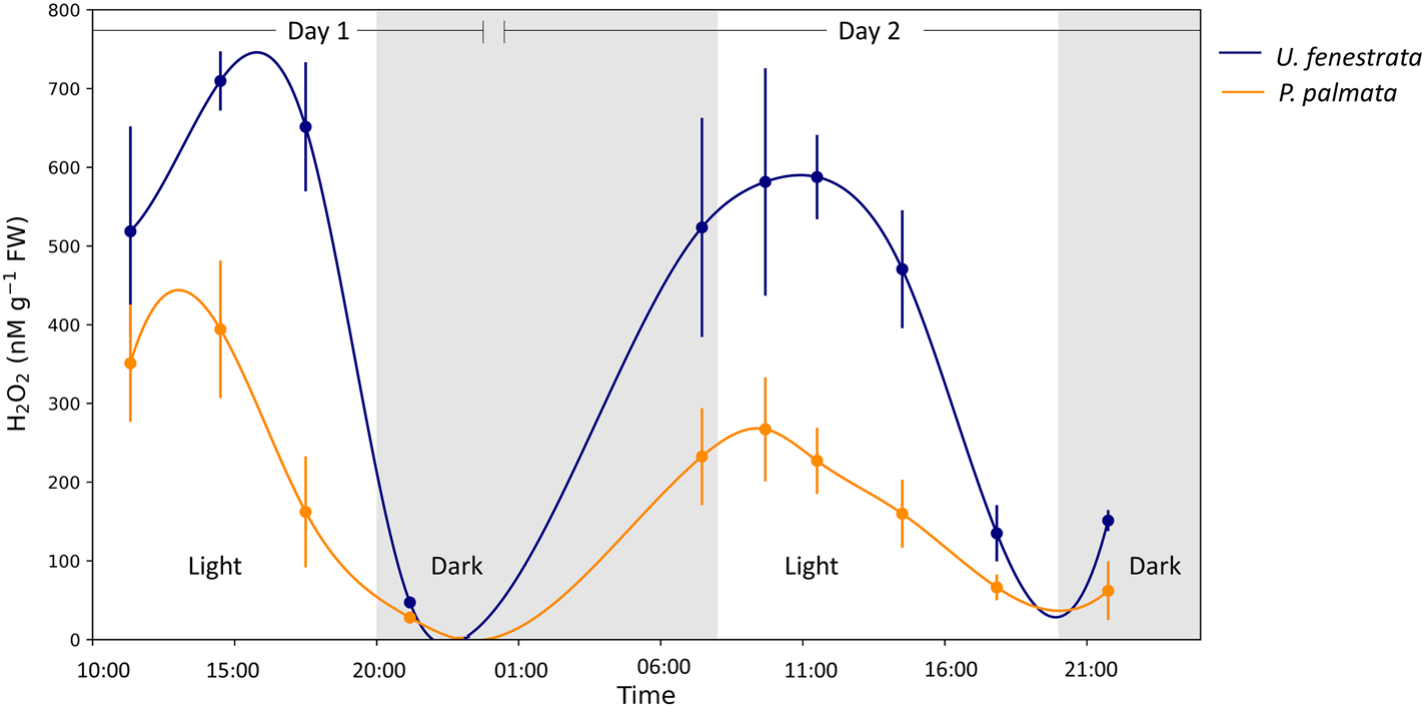
Diel H_2_O_2_ concentrations. H_2_O_2_ concentrations in seawater incubations of *U. fenestrata* (blue) and *P. palmata* (gold), shown in nM normalized per gram fresh weight of seaweed in the incubation. Data points display the average across four biological replicates, and the error bar represents ± 1 SD. Incubations of both species were done at 9 °C under 100 μmol m^2^ s^−1^ light and began at 8:00. Tabulated data available in Table S1. Control tanks without seaweed in Supplemental Fig. S1.

There were considerable fluctuations in the H_2_O_2_ concentrations in incubations of *U. fenestrata* and *P. palmata* over the course of the day (see Fig. 1). The H_2_O_2_ levels in the incubations of *U. fenestrata* were consistently higher than those for *P. palmata.* For both species, H_2_O_2_ concentrations were at a maximum at 14:00 on the first day, and around 11:00 on the second day. The highest concentration in *U. fenestrata* incubations on the first day was 710 (± 38) nM g^−1^ FW, while the maxima in *P. palmata* was 394 (± 87) nM g^−1^ FW. A dramatic decrease in H_2_O_2_ was observed prior to the onset of dark hours (set at 20:00) for both species with concentrations dropping to background H_2_O_2_ levels. In contrast, H_2_O_2_ concentrations in seaweed-free (control) tanks ranged only from 51 to 70 nM (see Figure S1).

The results of one-way analysis of variance (ANOVA) tests indicate that H_2_O_2_ concentrations in *U. fenestrata* and *P. palmata* are significantly different from one another as well as from control treatments not containing seaweeds across all timepoints apart from the measurement made at 21:10 on the first day of incubation (Table S2). At this timepoint, the H_2_O_2_ in the *P. palmata* and the *U. fenestrata* incubations are not significantly different from that of the control aquaria (Control – *Palmaria*, *P* = 0.0525; Control – *Ulva*: *P* = 0.776).

**Figure 2:**
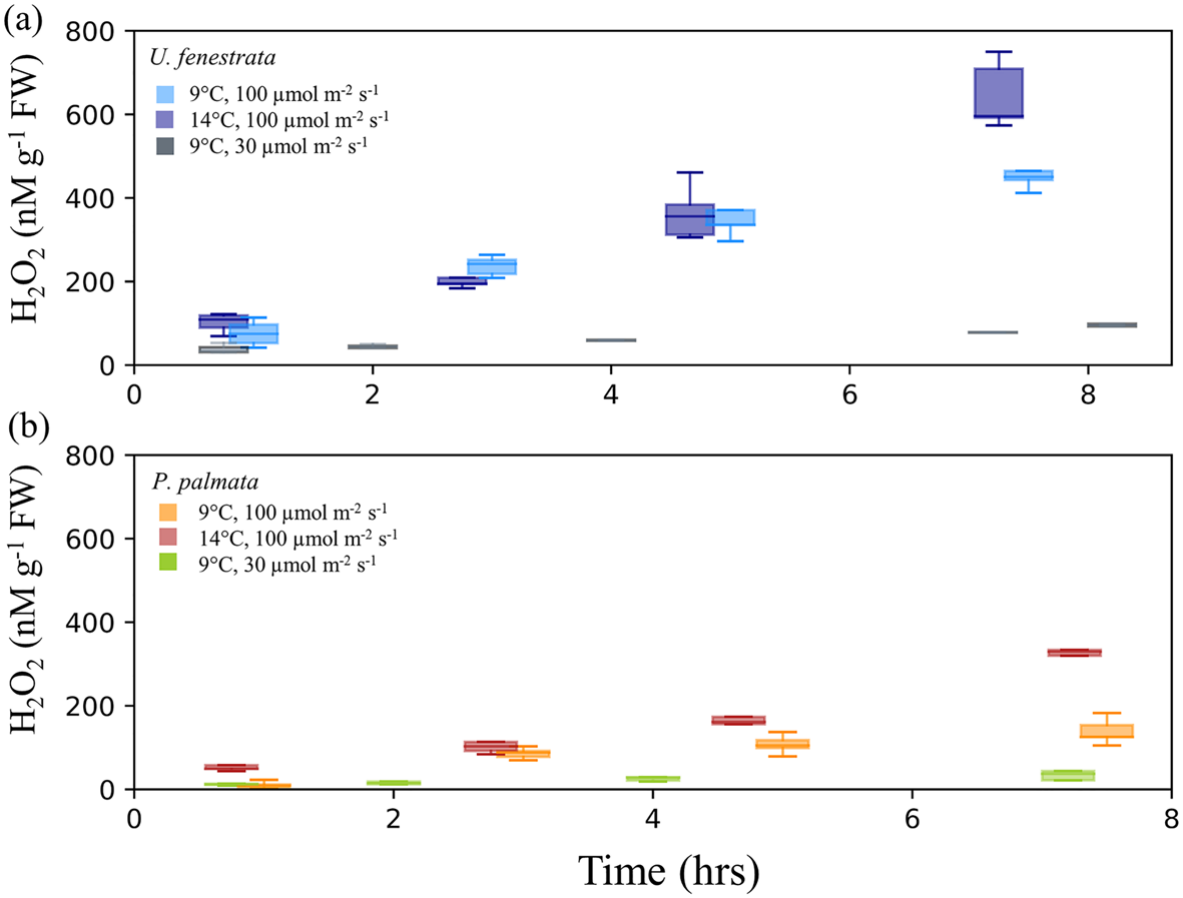
Effect of light and temperature on H_2_O_2_. Seawater H_2_O_2_ concentrations (shown as boxplots) over time for incubations of (**a**) *U. fenestrata* and (**b**) *P. palmata*. The three conditions were: (1) 9 °C and 100 μmol m^−2^ s^−1^ light, (2) 14 °C and 100 μmol m^−2^ s^−1^ and (3) 9 °C and 30 μmol m^−2^ s^−1^. For each experimental condition there were five biological replicates. Tabulated data available in Tables S3, S4, and S5.

Seaweeds grown in aquaculture are exposed to natural changes in temperature and light that occur over the course of a day or season. To better understand how shifts in these environmental conditions influence the production and decay of H_2_O_2_ we incubated individuals of *U. fenestrata.* and *P. palmata* at varying temperatures and light conditions (Fig. 2). In all experiments, except for the low light condition, H_2_O_2_ concentrations in the seawater of *U. fenestrata.* and *P. palmata* increased steadily with time. Experiments conducted under low light conditions (30 μmol m^−2^ s^−1^) did not lead to notable increases or decreases in H_2_O_2_ throughout the time-series for either species. We observed higher H_2_O_2_ concentrations in the aquaria of all *P. palmata* individuals, and all but one *U. fenestrata* when incubated at 14 °C in comparison to incubations at 9 °C.

A control tank consisting of seawater without macroalgae incubated under the same temperature and light conditions as the macroalgae incubations (9 °C control = 40.24 ± 0.96 nM; 14 °C control = 43.5 ± 2.01 nM; low light control = 39.91 ± 0.51 nM) showed no significant differences between treatment (*P* = 0.0525), nor across timepoints (*P* = 0.3255). Across the experimental timeline, *U. fenestrata* and *P. palmata* incubations at 14 °C and 9 °C were significantly different from one another (*P* < 0.0001). In contrast, between hours 2 and 8, the H_2_O_2_ in incubations of *U. fenestrata* and *P. palmata* under low light (30 µmol m^−2^ s^−1^, 9 °C) were not statistically distinguishable from each other, or from the seawater controls without seaweed. Results of the Student’s T-Tests comparing experimental treatments are summarized in Table S6.

### Effects of temperature, grazing, and desiccation on H_2_O_2_ concentrations

**Figure 3:**
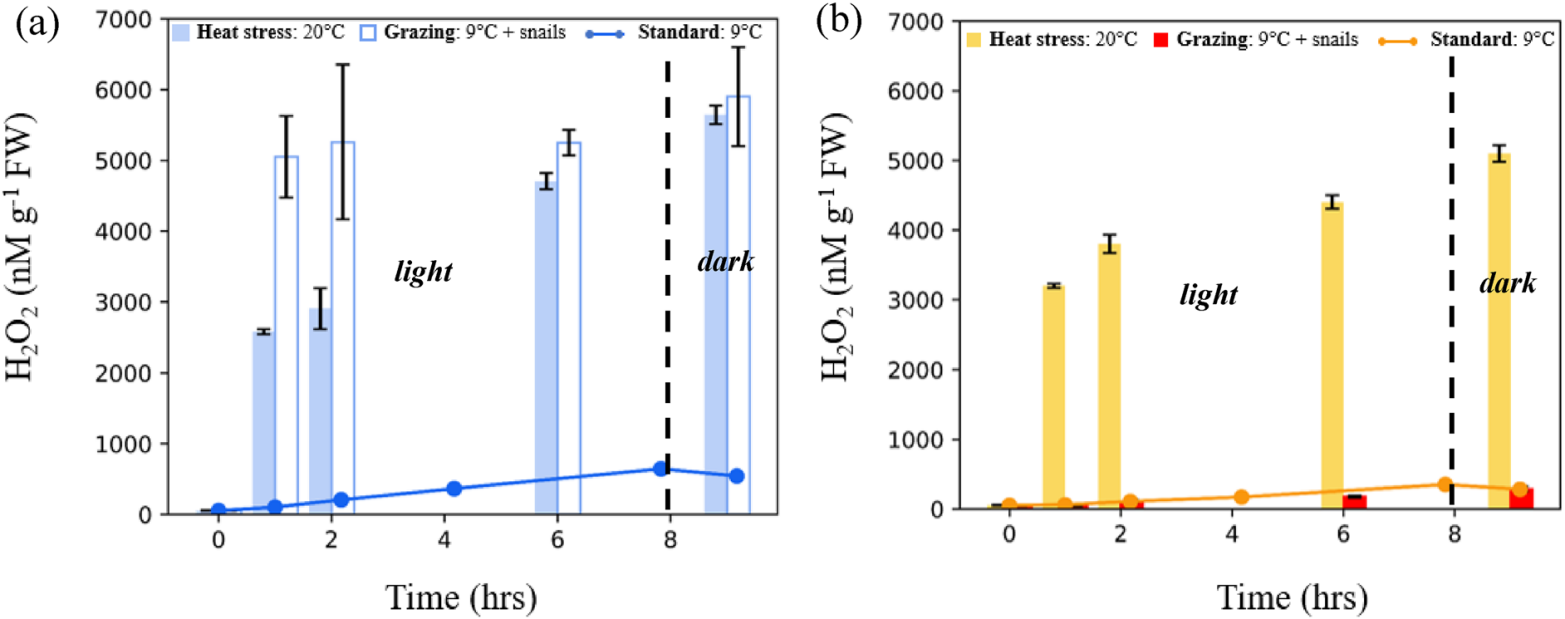
Effect of grazing and temperature on H_2_O_2_. The evolution of H_2_O_2_ in closed-top incubations of (**a**) *U. fenestrata* and (**b**) *P. palmata* over a period of approximately 9 h under three different conditions: standard (9 °C), acute heat stress at 20 °C, and when exposed to snails at 9 °C (not shown for *P. palmata*). The last hour of the incubation was in the dark. Incubations were done under 100 μmol m^−2^ s^−1^ light. The error bars show the ± 1 SD between biological replicates. Tabulated data available in Table S8.

To test the hypothesis that substantially elevated H_2_O_2_ concentrations will be induced by conditions in which the seaweeds experience stress, we examined responses to acute heat shock, grazing, and desiccation. As seen in Fig. 3, incubation at 20 °C led to far higher H_2_O_2_ concentrations in seawater compared to the standard condition at 9 °C. After an hour of incubation, the seawater H_2_O_2_ for *U. fenestrata* was 2853 (± 35) nM g^−1^ FW, amounting to a roughly 26-fold increase over the 109 (± 19) nM g^−1^ FW of the seawater in aquaria with individuals incubated under standard conditions. Similarly, incubations of *P. palmata* at 20 °C led to the accumulation of far higher H_2_O_2_ in seawater over the period of an hour than when grown under standard conditions (3200 in comparison to 50 nM g^−1^ FW). Across the experimental timeline, H_2_O_2_ in the seawater in the elevated temperature condition incubations increased steadily reaching 5,644 (± 128) nM g^−1^ FW and 5,200 (± 120) nM g^−1^ FW for *U. fenestrata* and *P. palmata,* respectively, after 9 h of incubation.

To evaluate the effect of grazing pressure, we grew individuals in the presence of herbivorous snails (*L. littorea*). Seawater in incubations where *U. fenestrata* individuals were exposed to the snails showed a spike in H_2_O_2_ levels within the first hour of incubation, reaching levels higher than under heat stress. In contrast to the observations of the temperature stress experiment, however, H_2_O_2_ concentrations were drastically elevated after an hour of incubation and remained relatively constant across the 9 h incubation timeline, increasing from 5048 (± 577) nM g^−1^ FW to 5899 (± 699) nM g^−1^ FW. Of the three experimental conditions, the most variability between biological replicates was seen in the grazing experiment, and the least was observed under the heat stress condition.

For all timepoints after one hour of incubation, the heat and grazing conditions resulted in H_2_O_2_ concentrations in *U. fenestrata* that were significantly different (*P* < 0.001) from the standard condition (Table S6). Similarly, the *P. palmata* heat treatment is significantly different (*P* < 0.001) from the H_2_O_2_ in the standard condition incubations. In contrast, the presence of *L. littorea* did not yield significantly different H_2_O_2_ in *P. palmata* incubations from that in the standard condition. By the end of the nine hours of incubation, the H_2_O_2_ levels in grazing and heat stress experiments were not statistically significant for *U. fenestrata* (*P* = 0.2333). The pattern of H_2_O_2_ accumulation in heat stress experiments of *U. fenestrate* and *P. palmata* was quite similar, but the total concentrations of H_2_O_2_ were still significantly different between the species at most timepoints.

**Figure 4:**
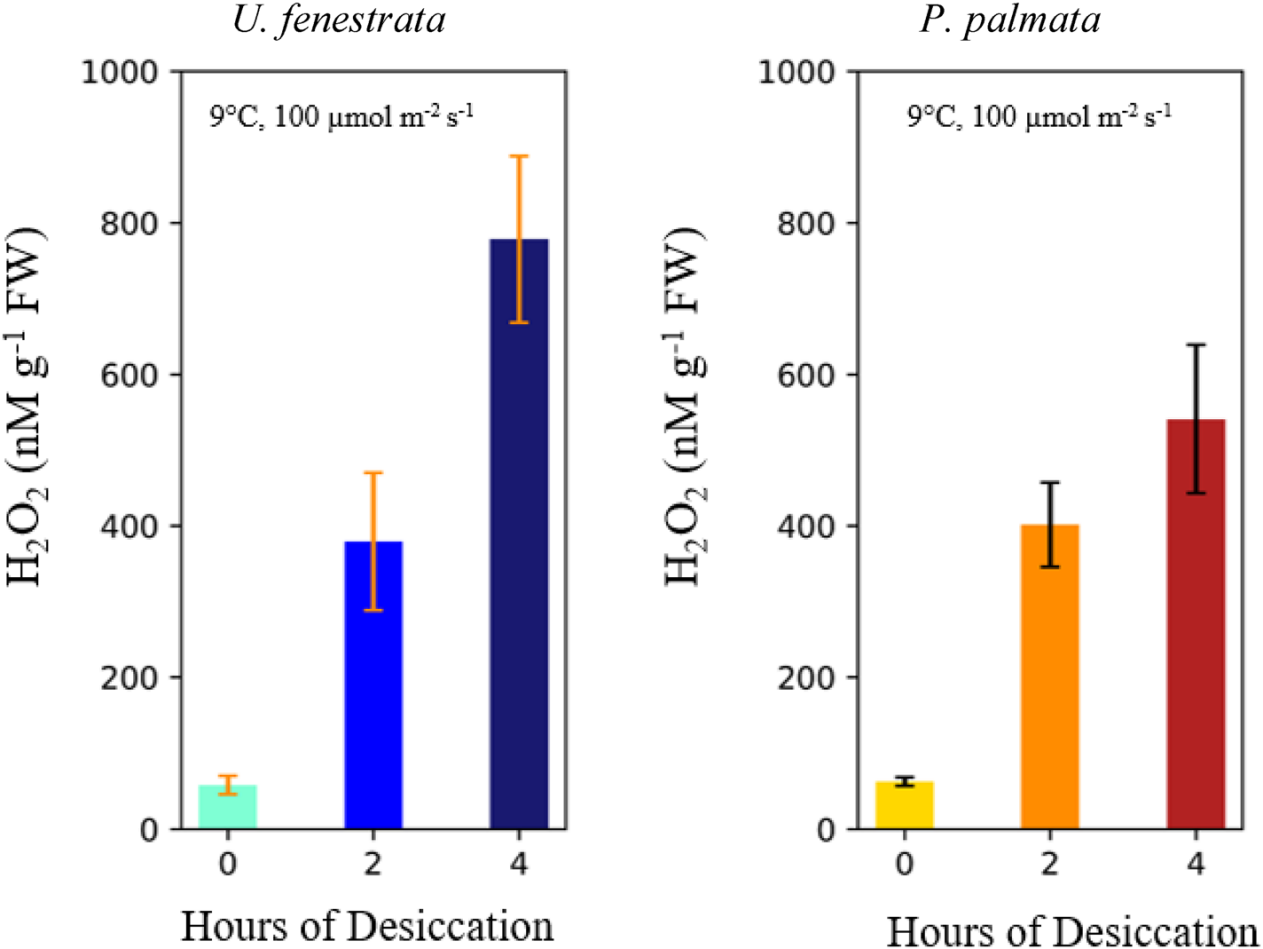
Effect of desiccation on H_2_O_2_. A comparison of seawater H_2_O_2_ concentrations after introduction of seaweeds that had not been desiccated, desiccated for 2 h, and desiccated for 4 h. Data for *U. fenestrata* shown in the graph on the left*, and P. palmata* on the right*.* All incubations were done under 100 μmol m^−2^ s^−1^ light. The error bars show the ± 1 SD of two biological replicates. Tabulated data available in Table S10.

The influence of desiccation on H_2_O_2_ concentrations was studied by removing seaweed from water for 2- or 4-h periods and then rehydrating them. As seen in Fig. 4, the seawater H_2_O_2_ levels measured 2 min after the introduction of seaweed was notably higher for incubations in which the individuals had been desiccated and scaled with the length of desiccation. In contrast to heat stress and grazing (Fig. 3), however, desiccation led to a far smaller shift from baseline H_2_O_2_ levels.

### Bromoform production

**Figure 5:**
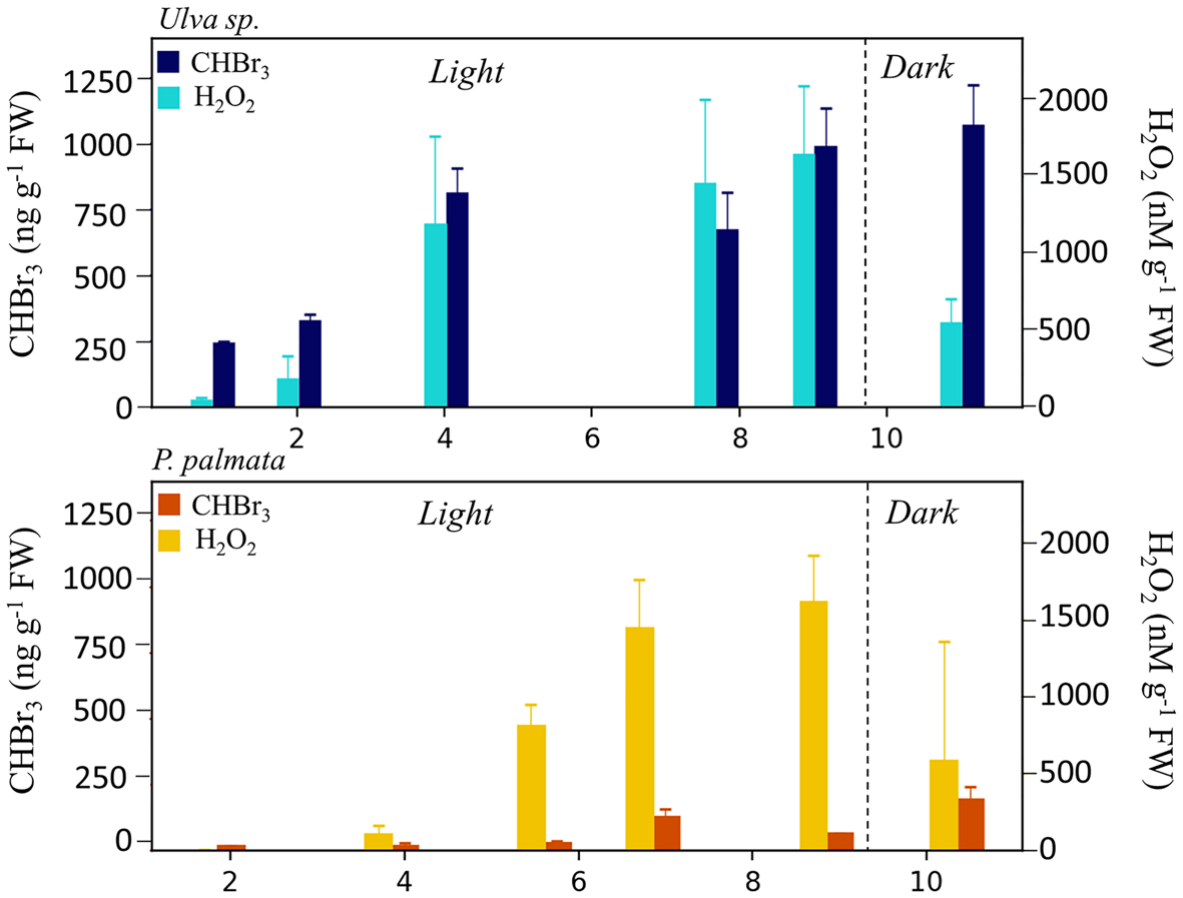
Time-series data of H_2_O_2_ and CHBr_3_ from closed-top incubations of *U. fenestra* (top) and *P. palmata* (bottom) at 9 °C in the presence of 130 μmol m^2^ s^−1^ light. The error bars show the ± 1 SD across three biological replicates. Tabulated data available in Table S7.

Seawater concentrations of CHBr_3_ in closed-top incubations of *U. fenestrata* increased from 244 to 1067 ng g^−1^ FW over the course of an 11-h period (Fig. 5). The corresponding H_2_O_2_ concentrations in the incubation waters increased from 44 to 1645 nM g^−1^ FW while the seaweeds were exposed to light (~ 1 h to 8.7 h of incubation), but dropped during the timepoint after the lights were turned off. In contrast to *U. fenestrata.* the seawater in which *P. palmata* was grown accumulated far less CHBr_3_ over time, ranging between 20 and 200 ng g^−1^ FW, and displays a general trend of increase. A similar pattern to *U. fenestrata* incubations, however, was observed for the seawater H_2_O_2_ concentrations, with an increase from approximately 3.5 to 1615 nM g^−1^ FW in the presence of light, and a drop to 588 nM g^−1^ FW after an hour in the dark.

**Figure 6:**
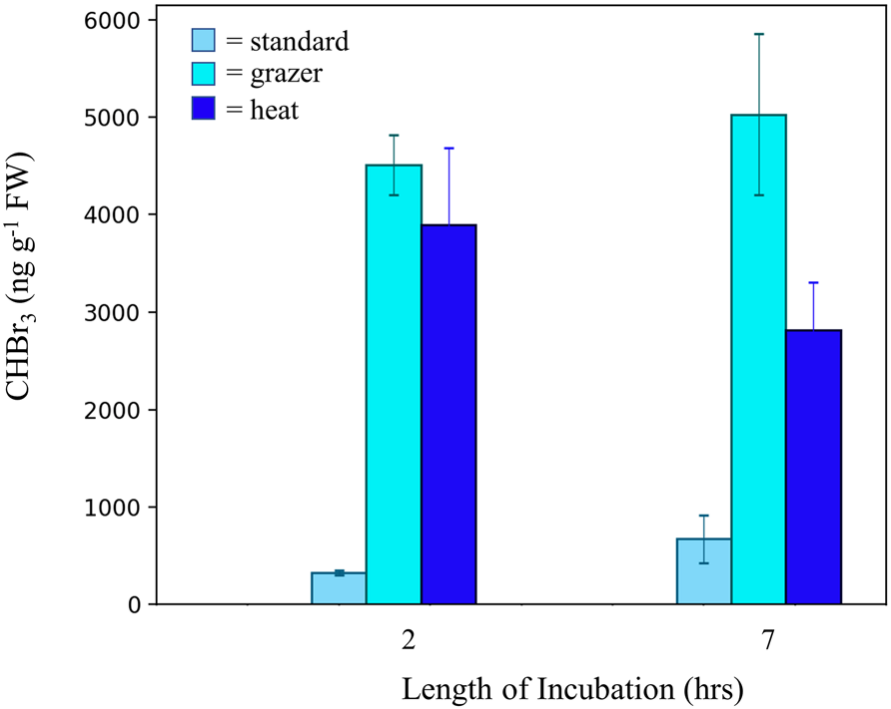
Seawater bromoform concentrations accumulated in incubation jars of *U. fenestrata* under standard conditions (set as 9 °C, under heat stress at 20 °C, and in the presence of snails at 9 °C. All incubations were exposed to 100 μmol m^−2^ s^−1^ light for the duration of the experimental timeline. The error bars show ± 1 SD of analytical and biological replicates. Tabulated data available in Table S11.

Incubation of *U. fenestrata* under both heat and grazing stress also greatly increased the concentrations of CHBr_3_. Seawater concentrations of CHBr_3_ in jars of *U. fenestrata* grown at 20 °C had the most CHBr_3_ (Fig. 6) of any condition at each timepoint and displayed an increase with length of time of incubation. Across biological replicates this resulted in CHBr_3_ concentrations of 5030 (± 826) ng g^−1^ FW after slightly over 6 h. *U. fenestrata* incubated with snails to induce a grazing pressure were also characterized by significantly elevated seawater CHBr_3_ conditions, but unlike in the heat stress condition later incubations (6.3 h timepoint) showed a decline in bromoform from incubation jars sampled earlier (2.7 h). In contrast, heat stress did not lead to significantly increased CHBr_3_ concentrations in incubation waters of *P. palmata* (after a 7 h incubation the CHBr_3_ concentrations (nM g^-1^ FW) were 57.53 ± 18.83 under standard conditions, 55.89 ± 14.84 under heat stress, and 60.12 ± 21.56 under grazing stress)*.*

## Discussion

Here we show that *U. fenestrata* and *P. palmata* produce H_2_O_2_ under ambient environmental conditions in the absence of stress (Fig. 1). These findings are in line with previous studies of a wide range of organisms, including a broad diversity of macroalga^26–28^. The enzymatic production of H_2_O_2_ in the absence of stress is linked to a suite of physiological processes in organisms, including cell signaling, cell differentiation, and nutrient acquisition. As such, we expected the basal levels of H_2_O_2_ within the seaweeds here to have natural fluctuations in response to physiological activity and circadian rhythms.

Indeed, we observed diel patterns in H_2_O_2_ concentrations in incubations of both *U. fenestrata* and *P. palmata*, ranging from 28 to 710 nM g^−1^ FW (see Fig. 1). Concentrations were generally highest in the morning to early-afternoon and decreased around 15:00–16:00. A similar trend in H_2_O_2_ but measured in greenhouse material of *Ulva rigida* over a 24-h period, and under different experimental conditions, was observed by Collén et al.^48^. As control incubations containing seawater without added algae did not show similar magnitudes to the seaweed incubations or significant variations with time (ranging only from 35 to 80 nM, see Fig. S1 and S2), we ascribe the observed H_2_O_2_ pattern over the two-day timeline to the seaweed and associated microorganisms, and not to abiotic or planktonic microbe production pathways.

The aqueous dynamics of H_2_O_2_ surrounding the seaweeds is a function of cumulative production and decay processes. Considering the ability of H_2_O_2_ to diffuse through cell membranes, the origin of H_2_O_2_ could be either intracellular or extracellular production pathways. To balance H_2_O_2_ generation and maintain healthy levels of ROS, seaweed also possesses antioxidative enzymes which scavenge H_2_O_2_^34^. A strategy of the antioxidant response that is particularly strong in seaweeds is the production of halogenated hydrocarbons^49^. Previous studies have shown that apart from the seaweed itself, microbial communities (including bacteria, fungi, and spores of marine invertebrates) associated with the seaweed surface^50–52^ can influence ROS dynamics in the external milieu. Species-specific differences in the relative strength of these production and decay pathways may account for the different, and generally higher concentrations of H_2_O_2_ in incubations of *U. fenestrata* than *P. palmata.*

The shift from increasing to decreasing H_2_O_2_ concentrations during our 2-day incubations signals the increasing strength of decay processes relative to production (Fig. 1). This change doesn’t coincide directly with the light–dark cycle (light from 8:00 to 20:00) in our incubations, but the higher H_2_O_2_ concentrations under light suggest that light-dependent processes are a primary driver of H_2_O_2_ formation. This could occur through photosynthetic generation of NADPH which is then used as a substrate by transmembrane NADPH oxidases (NOX /DUOX) enzymes, via the Mehler reaction, or some combination. The importance of light in driving baseline H_2_O_2_ dynamics was further supported by the observation of minimal accumulation of H_2_O_2_ under lower light conditions (30 μmol m^−2^ s^−1^) (Fig. 2).

Macroalgae in marine systems will experience a range in temperature and light conditions across growth seasons. For instance, sea-based aquaculture of *P. palmata* and *U. fenestrata* in northern Europe exposes seaweeds to natural variations in light availability, and a range of temperatures (~ 0.5 °C to 19 °C) throughout the year. To test for the response of H_2_O_2_ to small shifts in seawater temperature we incubated individuals at 14 °C (Fig. 2). For most biological replicates of both *U. fenestrata* and *P. palmata* this led to higher accumulations of H_2_O_2_ in the seawater after 5 h of incubation. One possible explanation for this is an increase in gross primary productivity, which has been recognized for many algal species at higher temperatures^53,54^.

We hypothesized that exposure to stressors would lead to significantly elevated concentrations in the seawater H_2_O_2_, that were distinguishable from natural fluctuations. Our results demonstrate drastic differences in the dynamics of seawater H_2_O_2_ when *U. fenestrata* and *P. palmata* were exposed to acute heat stress (20 °C), and grazing pressure by snails (Fig. 3a,b). Herbivory and wounding are known to lead to oxidative bursts in plants^23,55^ a response thought to help sessile organisms fend off attackers based on observations of elevated H_2_O_2_ in seawater leading to decreased grazing rates^36^. The primary source of ROS in the oxidative burst response is believed to be transmembrane NOX enzymes that coupled the oxidation of intracellular NADPH to the extracellular reduction of oxygen to superoxide and H_2_O_2_^56^. As previously seen in experiments with *U. lactuca*^57^, we observed that the snail *L. littorea* consumed *U. fenestrata*, leading to a significant increase in H_2_O_2_ levels*.* In earlier studies, McDowell et al., (2014, 2015) found that ROS production upon wounding depends on the photosynthetic electron transport chain^32,33^. We observed a continued increase in seawater H_2_O_2_ in incubations of *U. fenestrata* in the dark (see Fig. 4a), suggesting that photosynthetic activity is not requisite for H_2_O_2_ production. However, since these measurements were made just 1.2 h after dark, there may have been continued diffusion out of the plant, or lagging H_2_O_2_ production, and thus we cannot definitively rule out photosynthetic activity as a possible source of the observed dark H_2_O_2_ at this time.

There was no significant difference in seawater H_2_O_2_ concentrations when *P. palmata* was exposed to snails (*L. littorea)* from those under standard conditions (without grazers) (Fig. 3), which corresponded to the lack of observed grazing on and wounding of this species. Incubation at higher temperatures (20 °C) led to a considerable increase in H_2_O_2_ in the seawater, with elevated production also under dark conditions. Although H_2_O_2_ concentrations in the heat stress incubations were initially lower than under grazing pressure for *U. fenestrata,* the difference in response between the stress conditions became less notable after 6 h, perhaps pointing to a threshold H_2_O_2_ stress level. Overall, these findings highlight that light levels are a dominant factor for H_2_O_2_ production fluctuations under ambient conditions but become less influential in setting H_2_O_2_ levels in the presence of heat and grazing stress.

Seaweeds may produce CHBr_3_ through the uptake and subsequent oxidation of bromide (Br^−^) from seawater with H_2_O_2_. This reaction is catalyzed by extracellular vanadium haloperoxidases (V-HPOs)^43^. Building on previous work demonstrating CHBr_3_ in incubation studies of various species of macroalgae^58–60^, we set out to examine possible covariations between CHBr_3_ and natural H_2_O_2_. Our experiments show that the seawater in which *U. fenestrata* were incubated displayed an increase in CHBr_3_ concentrations with incubation time in the light that tracked H_2_O_2_, while in the *P. palmata* aquaria the CHBr_3_ concentrations did not rise considerably. Since *P. palmata* do possess vanadium-containing bromoperoxidases (V-BrPO)^61^, it is possible that H_2_O_2_ was preferentially used for the oxidation of other halides (I^−^, or Cl^−^), also catalyzed by V-BrPO, or the production of brominated organic compounds other than CHBr_3_. However, this would contradict previous studies which found that CHBr_3_ was the dominant halocarbon emitted by *P. palmata*^62,63^. For *U. fenestrata*, the relationship between seawater CHBr_3_ and H_2_O_2_ concentrations under standard conditions can be captured by a linear trend (R^2^ = 0.902) (see Fig. S2), suggesting that species-specific predictions of total CHBr_3_ production could be made with knowledge of surrounding seawater H_2_O_2_ concentrations in seaweed farms. Further experiments are required to elucidate the mechanisms which drive these species-specific variations in CHBr_3_ production under different conditions.

The increased production of CHBr_3_ has been linked to stress conditions such as nutrient depletion, and shifts in salinity, and temperature^40,41^. Confirming previous studies, we found that grazing and heat stress led to far greater production of CHBr_3_ in incubations of *U. fenestrata* (Fig. 6)*.* The drop in CHBr_3_ in the grazer incubation between 2 and 6 h of incubation time could reflect biological variability, or absorptive or degradative processes occurring in the seawater. The significantly elevated CHBr_3_ concentrations in seawater that we witnessed for *U. fenestrata* grown under stress conditions suggests that CHBr_3_ could act as a secondary monitoring target in aquaculture farms of certain species. Beyond the production of CHBr_3,_ the elevated levels of ROS that we expect to accompany dense macroalgal growth may lead to other changes in the local coastal environment, for instance through alterations in the carbon flow, and redox state of metals.

**Figure 7:**
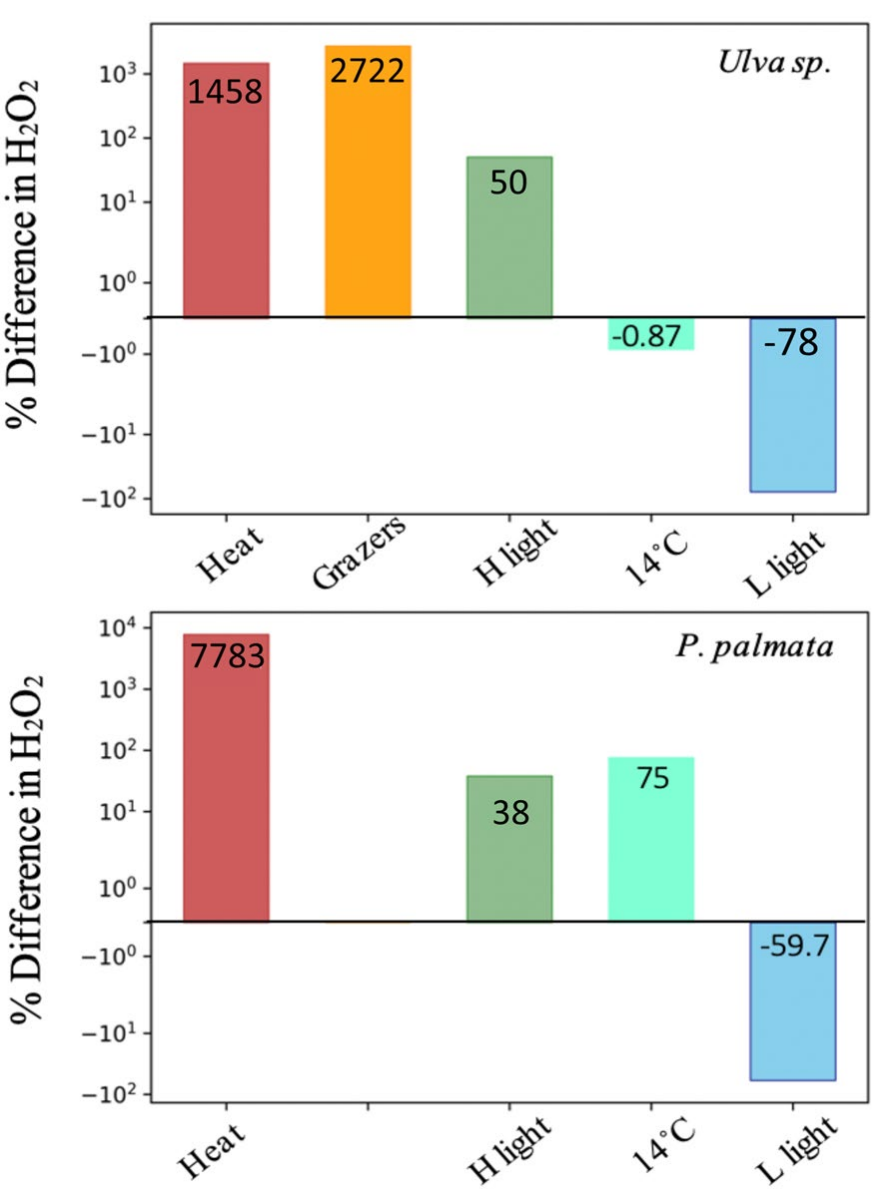
Percent difference in H_2_O_2_. Bars show the percent difference between stress and healthy treatments in aqueous H_2_O_2_ (shown on a logarithmic y-axis) after two hours of incubation in each experimental condition (listed on x-axis) relative to standard conditions of 9 °C under 100 μmol m^−2^ s^−1^ light. Heat: 20 °C, 100 μmol m^−2^ s^−1^ light; Grazers: presence of snails (*Littorina littorea),* 100 μmol m^−2^ s^−1^ light; H light (high light): 9 °C; 130 μmol m^−2^ s^−1^ light, 14 °C: 14 °C, and 100 μmol m^−2^ s^−1^ light; and L light (low light): 9 °C, and 30 μmol m^−2^ s^−1^ light.

A useful indicator of health for seaweed monitoring necessitates a marker molecule that is broadly applicable, reliably able to discern between standard and stressed conditions, and be readily measurable. Our data show that there are species-specific differences in the magnitude of H_2_O_2_ produced under varying temperature (9 to 14 °C) and light regimes (30,100, 130 μmol m^−2^ s^−1^) that may occur during natural diel or seasonal cycles. However, as seen in Fig. 7, exposure to heat stress and grazing yield differences in seawater H_2_O_2_ concentrations (1458 and 2722%) which are orders of magnitude larger than shifts seen in response to conditions falling within the expected natural environmental range. Elevated H_2_O_2_ concentrations were visible within an hour of exposure to the tested stressor, which suggests that monitoring aqueous H_2_O_2_ levels could serve an early indicator of stress conditions, and thus provide a rapid, and low-cost method to improve the biosecurity of sustainable seaweed farming in the future. We observed differences in H_2_O_2_ and CHBr_3_ dynamics of *U. fenestrata* and *P. palmata* underlining the importance of characterizing expected threshold H_2_O_2_ levels for each specific species that is being farmed. Future studies investigating time-series H_2_O_2_ and CHBr_3_ levels under varying stress and disease conditions, and field-based studies to characterize the physical effects and natural seawater concentrations will help develop the robustness of H_2_O_2_ as a monitoring method. Ultimately, the development of low-cost deployable H_2_O_2_ and CHBr_3_ sensors could provide a promising strategy for monitoring the health of seaweeds in aquaculture farms.

## Methods

### Algal strains and culture conditions

This study was conducted with individuals of *P. palmata* and *U. fenestrata.* that were removed from aerated indoor cultivations at Tjärno Marine Laboratory. The original specimens for the cultivations were collected in the vicinity of the lab (58° 50.257' N 11° 2.570′ E (WGS84 DDM) for *Palmaria*, and 58° 52.664′ N 11° 6.804′ E for *Ulva*). The *U. fenestrata* has been in cultivation for several generations (approximately for 1 year). The *P. palmata* proliferated vegetatively for approximately a 6-month period.

The seaweeds were grown in 90 L tanks provided with a filtered (5 µm + UV) natural seawater without addition of nutrients in a flow through system (10–14 L h^−1^), where aeration provided water motion. The seaweeds were exposed to a 16:8 (L:D) cycle at an irradiance of 140 µmol photons m^−1^ s^−1^ (light source INDY 66 LED 60 W 4000 k 6000 lm). Both the temperature and salinity conditions in the greenhouse followed seasonal variations. Further conditions of the cultivations and methods for the molecular identification of the species are described in Toth et al.^64^.

### H_2_O_2_ temporal dynamics

To characterize aqueous H_2_O_2_ dynamics associated with the seaweeds *U. fenestrata* and *P. palmata*, we tracked the concentrations of H_2_O_2_ in incubations at an ecologically relevant temperature of 9 ± 0.8 °C and light levels of 100 μmol m^−2^ s^−1^ (12-h light and 12-h dark) over a 2-day diel cycle.

Seaweed individuals were incubated in open-top 1 L plastic aquaria containing 470 mL of filtered seawater. All aquaria were randomly distributed and placed on two shelves in a closed, temperature-controlled incubator. Lights were controlled from a timer operated from the top of the incubator to reach 100 μmol m^−2^ s^−1^ white light. For each species, 5 aquaria with biological replicates were set up, and compared with 2 controls (containing just seawater). Aquaria were removed from the incubator for a short period of time 1–2 min for H_2_O_2_ analyses and then immediately returned.

In all incubations (including those described in later sections), the pH of the seawater in each incubation vessel was measured at the start and end of each experiment but remained within the range of 8.12–8.23. At the end of each experiment the seaweeds in each incubation were blotted dry and weighed to obtain fresh weight data (grams). The weight of *U. fenestrata* in aquaria was between 3.2 and 4.5 g, and 5.2–6.7 g for *P. palmata.*

To test for differences in H_2_O_2_ dynamics resulting from small shifts in temperature and light, three experiments were setup on sequential days: a comparison of *U. fenestrata* vs *P. palmata* at 9 °C under 100 μmol m^−2^ s^−1^, at 14 °C grown under 100 μmol m^−2^ s^−1^, and under low light conditions (30 μmol m^−2^ s^−1^) at 9 °C. Each experiment lasted approximately 8 h, during which the seaweeds were exposed to a constant light level (either 100 or 30 μmol m^−2^ s^−1^). The incubations began between 7:00 and 8:00 in the morning. For each species five biological replicates were set up, as described above for the 2-day experiments. All experiments were conducted in the same incubator.

### Effects of temperature, grazing, and desiccation on H_2_O_2_ concentrations

In heat stress experiments, the incubation temperature was set to 20 °C. This constitutes a temperature increase of ~ 11 °C from the standard cultivation, which is not likely to be realized in the natural environment but rather is meant to represent a scenario of acute stress. As in previous experiments, open-top 1 L plastic aquaria containing 470 mL of filtered seawater were prepared. To obtain the desired water temperature the aquaria were placed into the incubator 5 h prior to the start of the experiment. The seawater temperature was measured with a glass thermometer right before placing the seaweeds in to verify that the temperature was 20 °C (± 0.5 °C). Two aquaria of *U. fenestrata,* two aquaria of *P. palmata*, as well as two control aquaria that did not contain seaweeds were placed on the same shelf in the incubator under 100 μmol m^−2^ s^−1^ light. The experiments ran over slightly nine hours, the first eight hours of which were under light, and the last 1.2 h were in the dark (lights inside incubator toggled off on a timer). As in other experiments aquaria were briefly removed from the incubator for H_2_O_2_ analysis.

Grazing experiments were conducted in a similar manner as the heat stress experiments described above but at a temperature of 9 °C, and with the addition of three *L. littorea* snails in each experimental aquarium. The snails were collected from the shore right outside the lab and were kept for two days under running seawater before being used in experiments. Two aquaria were set up for each species respectively, as well as two controls containing seawater with snails but no seaweed.

In desiccation experiments, seaweed individuals were removed from long-term cultivation tanks and left out to dry under ambient room conditions (18 °C) for 2 or 4 h respectively before being submerged in plastic 1 L aquaria containing 470 mL of filtered seawater. Two minutes after the seaweeds were submerged, the H_2_O_2_ in the seawater was measured. A control was performed by removing the seaweed and directly placing it in the seawater (0 h of desiccation). Desiccation experiments were done in duplicates for each species.

### Bromoform production

For experiments conducted for contemporaneous CHBr_3_ and H_2_O_2_ measurements, individuals were incubated in screw-top glass mason jars filled to the brim with 470 mL of filtered seawater. A piece of aluminum foil was placed over the top of the jar with the dull side facing the inside of the bottle, and the lid was tightly screwed shut paying attention that no creases formed. The purpose of the aluminum foil is to eliminate contact between the incubation water and the lid which may lead to extraction of CHBr_3_ from the water.

Two experiments were done for contemporaneous CHBr_3_ and H_2_O_2_ analysis: a time-series experiment of both *U. fenestrata* and *P. palmata*, and a stress response experiment of *U. fenestrata.* In the time-series experiments, incubations were done at 9 °C and 130 μmol m^−2^ s^−1^ light. The first nine hours of the incubation were done in the presence of light, and the last two in the dark. The incubations were set up around 11:00 and dark conditions ensued at 20:00. Three incubation jars were set up per species per timepoint and were sacrificed at the end of the respective timepoint. Three controls containing just seawater but prepared in mason jars with aluminum foil lining as well were set up for each of two timepoints (1 h and 7.83 h incubation lengths). The jars were removed from the incubator at the time of measurement. Volatiles were sampled first (as described below), and then measured for H_2_O_2_. The same procedure was followed for volatile analysis in stress experiments of *U. fenestrata.* Sacrificed jars were prepared in duplicates for heat stress at 20 °C under 100 μmol m^−2^ s^−1^ light, and for grazing in which jars contained three *Littorina littorea* snails. These were compared with a standard condition in which individuals of *U. fenestrata* were incubated at 9 °C under 100 μmol m^−2^ s^−1^ light.

### Hydrogen peroxide analysis

Measurements of H_2_O_2_ were made with the use of commercially available hydrogen peroxide micro- and macro-sensors (WPI ISO HPO 2, HPO 100) connected to a free radical analyzer (TBR 4100). Sensor readings were monitored in real-time through LabScribe software operated from a laptop.

Four-point calibration curves were made at the start of every day relating a known concentration of H_2_O_2_ (nM) to a current (pA). Standard solutions were prepared with a 30% H_2_O_2_ solution (VWR Chemicals) in filtered seawater which was also used for the incubations. Care was taken to calibrate the sensor in solutions at the temperature of the experiment being run to avoid any temperature-related effects in measurement.

Incubation waters were analyzed for H_2_O_2_ concentration by submerging the tip of the sensor into the 1L aquarium or mason jar for a duration of approximately 1 min. To calculate the H_2_O_2_ concentration in the incubation water the current readings between 20 and 60 s were averaged, allowing the first 20 s for stabilization of the sensor. Between measurements in different incubation containers the sensor was briefly cleaned by swirling it in deionized water.

### Statistical analyses of H_2_O_2_ measurements

To determine whether experimental treatments (e.g. *Ulva* Heat Stress, *Palmaria* Grazing, *Ulva* Standard, *Palmaria* 14C, etc.) resulted in differences in H_2_O_2_ that were statistically significant (using a significance level of 0.05) from one another, one-way analysis of variance (ANOVA) were performed at each timepoint to examine the differences in treatment. If the timepoints of comparison did not exactly align for each treatment, values were interpolated to predict the H_2_O_2_ concentration at the chosen timepoint of examination. In situations where the results of the ANOVA revealed that some treatments were not significantly different (a *P* > 0.05), individual Student’s T-Tests were done to compare means between individual treatments at specific timepoints. Levene’s Tests were done to confirm equality in variances which is an assumption of a Student’s T-Test. All statistical analyses were done in the software program JMP. The results of the tests are given in the Supplementary Information.

### Volatile organic compound sampling and analysis

At each experimental timepoint, two samples of water for volatile analysis were removed from the sealed incubation jars (covered with aluminum foil between water and lid) and transferred to 40 mL volatile organic analysis (VOA) vials. Samples were acidified to a pH < 2 with approximately 530 uL of a 0.75 M HCl solution and stored in the cold (4 °C) and dark.

The halocarbon content of the sampled incubation seawater was analyzed within two weeks of collection using a purge and trap coupled with gas chromatography (Agilent 7890B) with electron capture detection (GC-ECD). The calculated maximum holding time for acid-preserved volatiles in water stored in 40 mL borosilicate vials was previously estimated at approximately 112 days^65^, which we are well under here. Volatiles were purged for 12 min with UHP nitrogen at a flow rate of 40 mL min^−1^. Upon completion of trapping, the sample was desorbed onto the column at 150 °C temp. The GC temperature was set at 45 °C during the injection and held for 5 min. The temperature was then ramped at 8 °C min^−1^ to 130 °C, at 12 °C min^−1^ to 150 °C and held for 2 min, and then at 15 °C min^−1^ until 180 °C and held for 2 min. Samples were investigated primarily for bromoform content, which was identified by retention time and comparison to standards. The concentration of bromoform was quantified using a standard curve prepared with a commercially available standard in methanol.

### Supplementary Information

Supplementary Information.

## Data Availability

Data available in tabulated form in the Supplementary Information.
