# Supplementary material for: Assessment of hydrogen peroxide as a bioindicator of stress in seaweed aquaculture
Source: Sci Rep. 2024 Jan 23;14:1956. doi: 10.1038/s41598-024-52182-5 (PMC10806984; doi:10.1038/s41598-024-52182-5)
Supplement: Supplementary file 1 — Supplementary Information. [file 41598_2024_52182_MOESM1_ESM.pdf]

Supplementary Information for Assessment of Hydrogen Peroxide as a Bioindicator of Stress in Seaweed Aquaculture

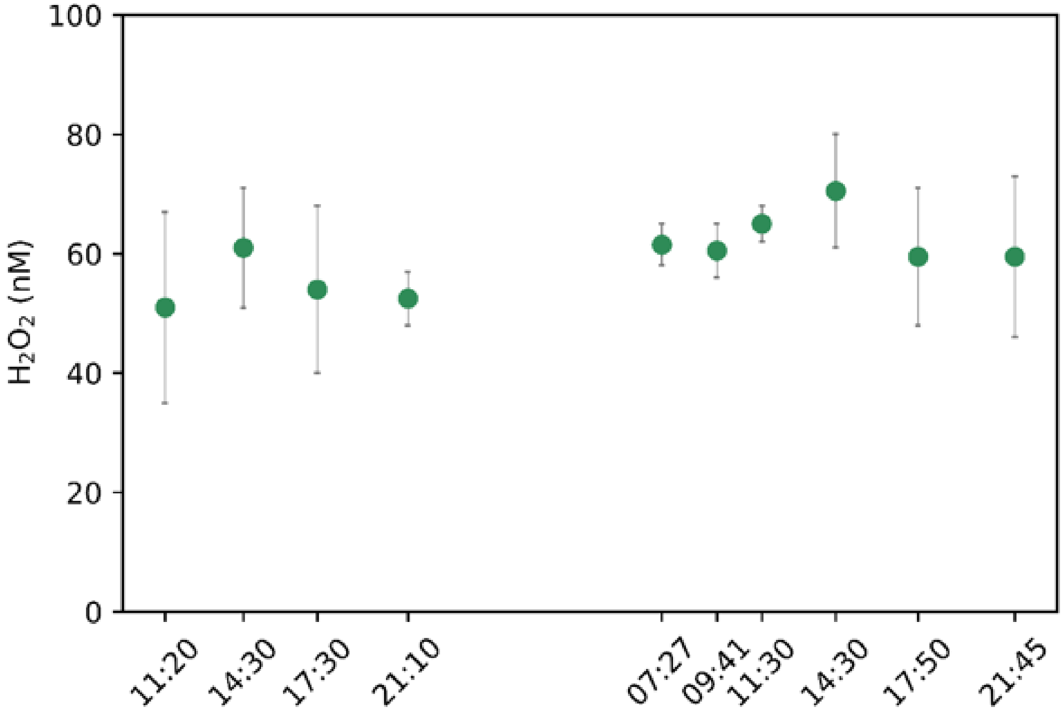

Fig S1. Variation in seawater H_2O_2 over a two-day incubation. Concentrations of H_2O_2 (nM) in incubations of seawater (without macroalgae) across a 2-day experiment. Incubations were done at 9°C under 100 μmol m^{-2} s^{-1}. Each data point represents the average of two controls, and the error bar shows the standard deviation.

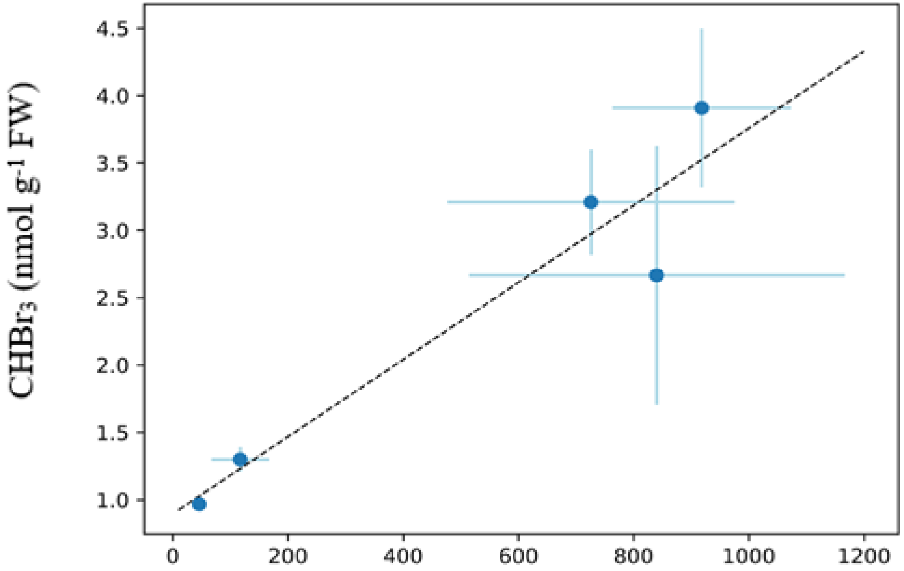

Figure S2. A linear regression between H_2O_2 and CHBr3 associated with *Ulva sp.* incubation at 9°C and 130 umol m^{-2} s^{-1}.

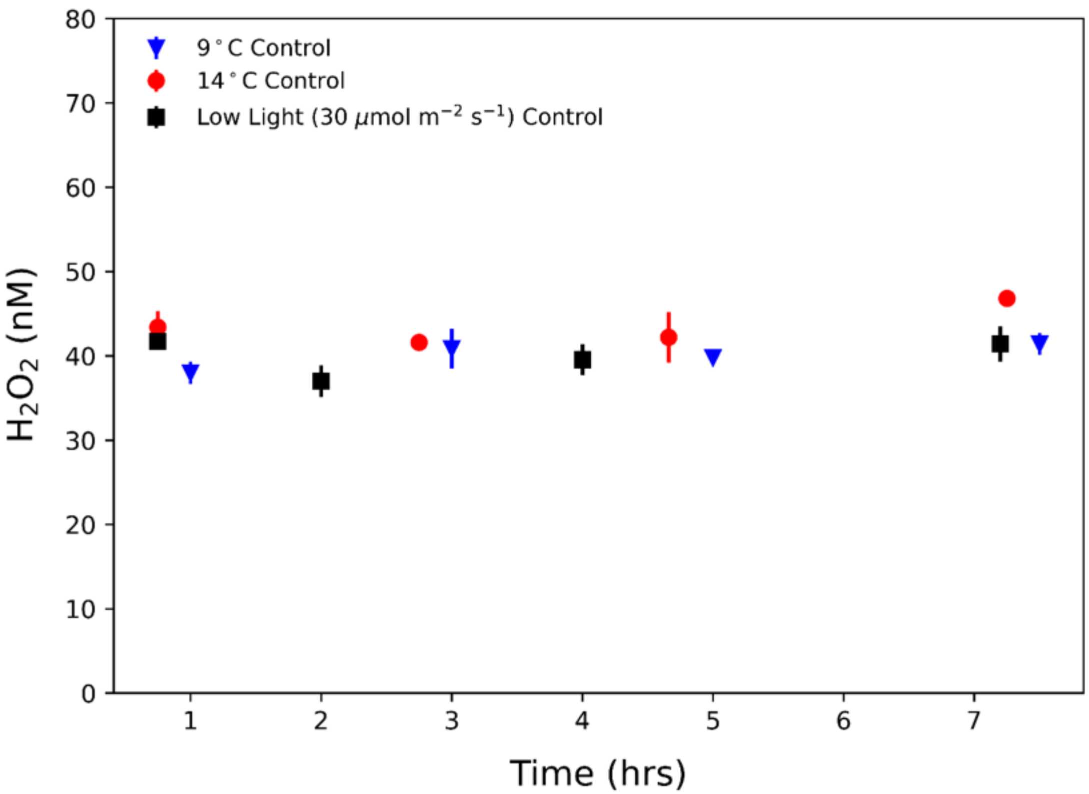

Fig S3. H_2O_2 (nM) in different treatments of seawater over seven hours.
Error bars show the standard deviation of two replicate incubations.

	H_2O_2 (nM g^{-1} FW)			
Time	*U. fenestra* Av. over Bio. Rep.'s	*U. fenestrata* Std. Dev of Bio. Rep.'s	*P. palmata* H_2O_2 (nM g^{-1} FW) Standard Dev of Bio. Rep.'s	*P. fenestra* H_2O_2 (nM g^{-1} FW) Standard Dev of Bio. Rep.'s
10/7/22 11:20	518.7	133	351.17	74.52
10/7/22 14:30	709.7	38	394.23	87.34
10/7/22 17:30	651.4	82	162.28	70.54
10/7/22 21:10	47.4	6.07	28.06	3.63
10/8/22 7:27	523.6	139.23	232.48	61.51
10/8/22 9:41	581.4	144.44	267.24	66.11
10/8/22 11:30	587.6	53.59	227.16	42.10
10/8/22 14:30	470.6	74.91	159.97	43.26
10/8/22 17:50	135.1	35.72	66.46	16.35
10/8/22 21:45	151.3	13.38	62.08	37.27

Table S1. H_2O_2 (nM g^{-1} FW) concentrations in seawater during incubations of individuals of *P. palmata* and *U. fenestrata* at 9˚C data under 100 umol m^2 s^{-1} light across a two-day experimental timeline. Data corresponding to Figure 1.

Time	ANOVA (P-Value)
11:20	.0003*
14:30	0.0142*
17:30	0.0069*
21:10	0.0649
07:27	<.0001*
09:41	.0075*
11:30	0.0054*
14:30	0.0142*
17:50	0.0070*
21:45	0.0104*

Timepoint 21:10	
Levene's Test	P = 0.3865
Student's T-Tests	
Control-Palmaria	0.0525
Ulva-Palmaria	0.0413*
Control - Ulva	0.776

Table S2. Tests for the presence of significant differences between treatments at individual timepoints. The table on the left shows the results of a one-way analysis of variance between H_2O_2 concentrations in incubations of *U. fenestrata* and *P. palmata* at 9°C under 100 µmol m^{-2} s^{-1} light for each timepoint. P values less than 0.05 are considered significantly different. Student's T-Tests were performed for timepoint 21:10 (shown in the table on the right) to determine which treatments were different.

9˚C												
	P. palmata **H_2O_2 (nM g^{-1} FW)**					***U. fenestra*** **H_2O_2 (nM g^{-1} FW)**					**Control**	
Time (hrs)	Bio. Rep. #1	Bio. Rep. #2	Bio. Rep. #3	Bio. Rep. #4	Bio. Rep. #5	Bio. Rep. #1	Bio. Rep. #2	Bio. Rep. #3	Bio. Rep. #4	Bio. Rep. #5	R1	R2
1	23.62	12.06	9.60	1.25	4.54	74.8	52.5	42.0	97.3	113.9	40.2	37.6
3	92.85	103.17	88.09	78.29	70.47	242.5	208.8	263.8	218.3	252.0	38.5	43.2
5	137.61	118.17	99.52	105.54	79.75	335.0	523.6	296.4	371.0	336.0	46.1	39.5
7.5	183.29	154.53	125.06	126.17	105.98	464.4	729.0	443.1	450.1	411.8	42.7	40.1

Table S3. H_2O_2 (nM g^{-1} FW) concentrations in seawater during incubations of individuals of *P. palmata* and *U. fenestrata* at 9˚C data under 100 umol m^2 s^{-1} light, as well as two control replicates of seawater containing no algal biomass.

14˚C												
	P. palmata **H_2O_2 (nM g^{-1} FW)**					***U. fenestra*** **H_2O_2 (nM g^{-1} FW)**					**Control**	
Time (hrs)	Bio. Rep. #1	Bio. Rep. #2	Bio. Rep. #3	Bio. Rep. #4	Bio. Rep. #5	Bio. Rep. #1	Bio. Rep. #2	Bio. Rep. #3	Bio. Rep. #4	Bio. Rep. #5	R1	R2
1	100.74	58.85	44.95	49.70	49.80	69.80	122.13	90.57	109.60	119.81	45.3	41.5
3	151.02	114.93	92.50	103.74	84.15	184.83	209.40	194.10	245.86	195.07	42.6	40.6
4.66	242.26	161.59	157.00	174.21	124.44	356.32	461.51	312.46	384.65	306.63	39.2	45.2
7.25	512.93	320.43	330.43	334.60	262.64	596.98	709.51	575.00	750.34	592.34	46.4	47.2

Table S4. H_2O_2 (nM g^{-1} FW) concentrations in seawater during incubations of individuals of *P. palmata* and *U. fenestrata* at 14˚C data under 100 m^{-2} s^{-1} light, as well as two control replicates of seawater containing no algal biomass, at 14˚C data under 100 umol m^{-2} s^{-1} light.

Low Light													
	P. palmata **H_2O_2 (nM g^{-1} FW)**					***U. fenestra*** **H_2O_2 (nM g^{-1} FW)**						**Control**	
Time (hrs)	Bio. Rep. #1	Bio. Rep. #2	Bio. Rep. #3	Bio. Rep. #4	Bio. Rep. #5	Time (hrs)	Bio. Rep. #1	Bio. Rep. #2	Bio. Rep. #3	Bio. Rep. #4	Bio. Rep. #5	R1	R2
0.75	46.80	12.80	14.56	9.51	11.50	0.75	33.89	30.05	31.12	42.74	52.90	40.9	42.5
2	41.16	15.13	19.27	12.23	12.48	2	50.48	28.98	40.52	44.24	46.54	38.9	35.1
4	60.79	29.04	28.57	21.44	19.57	4	60.72	58.07	58.65	73.20	53.30	37.7	41.4
7.2	80.75	37.41	44.61	22.83	22.36	7.2	79.06	69.65	78.18	92.52	77.58	39.3	43.5
						8.2	130.00	92.47	98.80	95.30	77.37	N/A	N/A

Table S5. H_2O_2 (nM g^{-1} FW) concentrations in seawater during incubations of individuals of *P. palmata* and *U. fenestrata* at 9˚C data under 30 umol m^{-2} s^{-1} light, as well as two control replicates of seawater containing no algal biomass, at 14˚C data under 30 umol m^{-2} s^{-1} light.

Ordered Differences Report

Level	- Level	Difference	Std Err Dif	Lower CL	Upper CL	p-Value
Ulva 14C	Palmaria 9C	92.16800	10.96724	69.8002	114.5358	<.0001*
Ulva 14C	Palmaria LL	83.34800	10.96724	60.9802	105.7158	<.0001*
Ulva 9C	Palmaria 9C	65.88600	10.96724	43.5182	88.2538	<.0001*
Ulva 14C	Ulva LL	64.24200	10.96724	41.8742	86.6098	<.0001*
Ulva 14C	Control	60.23200	9.88574	40.0699	80.3941	<.0001*
Ulva 9C	Palmaria LL	57.06600	10.96724	34.6982	79.4338	<.0001*
Palmaria 14C	Palmaria 9C	50.59400	10.96724	28.2262	72.9618	<.0001*
Palmaria 14C	Palmaria LL	41.77400	10.96724	19.4062	64.1418	0.0006*
Ulva 14C	Palmaria 14C	41.57400	10.96724	19.2062	63.9418	0.0007*
Ulva 9C	Ulva LL	37.96000	10.96724	15.5922	60.3278	0.0016*
Ulva 9C	Control	33.95000	9.88574	13.7879	54.1121	0.0017*
Control	Palmaria 9C	31.93600	9.88574	11.7739	52.0981	0.0029*
Ulva LL	Palmaria 9C	27.92600	10.96724	5.5582	50.2938	0.0161*
Ulva 14C	Ulva 9C	26.28200	10.96724	3.9142	48.6498	0.0228*
Control	Palmaria LL	23.11600	9.88574	2.9539	43.2781	0.0260*
Palmaria 14C	Ulva LL	22.66800	10.96724	0.3002	45.0358	0.0472*
Ulva LL	Palmaria LL	19.10600	10.96724	-3.2618	41.4738	0.0914
Palmaria 14C	Control	18.65800	9.88574	-1.5041	38.8201	0.0685
Ulva 9C	Palmaria 14C	15.29200	10.96724	-7.0758	37.6598	0.1731
Palmaria LL	Palmaria 9C	8.82000	10.96724	-13.5478	31.1878	0.4274
Control	Ulva LL	4.01000	9.88574	-16.1521	24.1721	0.6878

Timepoint 1

Ordered Differences Report

Level	- Level	Difference	Std Err Dif	Lower CL	Upper CL	p-Value
Ulva 9C	Ulva LL	337.5350	29.42354	277.444	397.6259	<.0001*
Ulva 9C	Control	330.2500	25.00520	279.183	381.3174	<.0001*
Ulva 14C	Ulva LL	329.4490	29.42354	269.358	389.5399	<.0001*
Ulva 14C	Control	322.1640	25.00520	271.097	373.2314	<.0001*
Ulva 9C	Palmaria LL	307.7520	27.74078	251.098	364.4062	<.0001*
Ulva 14C	Palmaria LL	299.6660	27.74078	243.012	356.3202	<.0001*
Ulva 9C	Palmaria 9C	264.2820	27.74078	207.628	320.9362	<.0001*
Ulva 14C	Palmaria 9C	256.1960	27.74078	199.542	312.8502	<.0001*
Ulva 9C	Palmaria 14C	200.5000	27.74078	143.846	257.1542	<.0001*
Ulva 14C	Palmaria 14C	192.4140	27.74078	135.760	249.0682	<.0001*
Palmaria 14C	Ulva LL	137.0350	29.42354	76.944	197.1259	<.0001*
Palmaria 14C	Control	129.7500	25.00520	78.683	180.8174	<.0001*
Palmaria 14C	Palmaria LL	107.2520	27.74078	50.598	163.9062	0.0006*
Palmaria 9C	Ulva LL	73.2530	29.42354	13.162	133.3439	0.0186*
Palmaria 9C	Control	65.9680	25.00520	14.901	117.0354	0.0131*
Palmaria 14C	Palmaria 9C	63.7820	27.74078	7.128	120.4362	0.0286*
Palmaria 9C	Palmaria LL	43.4700	27.74078	-13.184	100.1242	0.1276
Palmaria LL	Ulva LL	29.7830	29.42354	-30.308	89.8739	0.3195
Palmaria LL	Control	22.4980	25.00520	-28.569	73.5654	0.3754
Ulva 9C	Ulva 14C	8.0860	27.74078	-48.568	64.7402	0.7727
Control	Ulva LL	7.2850	26.85989	-47.570	62.1402	0.7881

Timepoint 3

Table S6. Results of Student T-Tests comparing the means between incubations of *U. fenestrata and P. palmata:* at 14°C and 9°C under 100 µmol $m^{-2}s^{-1}$, 9°C under 30 µmol $m^{-2}s^{-1}$, with control seawater incubations. The data used in these tests is tabulated in Tables S3-S5. Controls containing just seawater were set up under each respective experimental condition, however, as ANOVAs of control H_2O_2 by treatment (P=0.0525) and time (P=0.3255) were not significantly different, a singular average control value was used in these T-tests.

Ordered Differences Report

Level	- Level	Difference	Std Err Dif	Lower CL	Upper CL	p-Value
Ulva 9C	Ulva LL	202.0983	9.77696	182.183	222.0133	<.0001*
Ulva 9C	Palmaria LL	201.2816	10.21170	180.481	222.0822	<.0001*
Ulva 9C	Control	194.9300	9.20470	176.181	213.6794	<.0001*
Ulva 14C	Ulva LL	170.8703	9.77696	150.955	190.7853	<.0001*
Ulva 14C	Palmaria LL	170.0536	10.21170	149.253	190.8542	<.0001*
Ulva 14C	Control	163.7020	9.20470	144.953	182.4514	<.0001*
Ulva 9C	Palmaria 9C	150.5060	10.21170	129.705	171.3066	<.0001*
Ulva 9C	Palmaria 14C	127.8120	10.21170	107.011	148.6126	<.0001*
Ulva 14C	Palmaria 9C	119.2780	10.21170	98.477	140.0786	<.0001*
Ulva 14C	Palmaria 14C	96.5840	10.21170	75.783	117.3846	<.0001*
Palmaria 14C	Ulva LL	74.2863	9.77696	54.371	94.2013	<.0001*
Palmaria 14C	Palmaria LL	73.4696	10.21170	52.669	94.2702	<.0001*
Palmaria 14C	Control	67.1180	9.20470	48.369	85.8674	<.0001*
Palmaria 9C	Ulva LL	51.5923	9.77696	31.677	71.5073	<.0001*
Palmaria 9C	Palmaria LL	50.7756	10.21170	29.975	71.5762	<.0001*
Palmaria 9C	Control	44.4240	9.20470	25.675	63.1734	<.0001*
Ulva 9C	Ulva 14C	31.2280	10.21170	10.427	52.0286	0.0045*
Palmaria 14C	Palmaria 9C	22.6940	10.21170	1.893	43.4946	0.0335*
Control	Ulva LL	7.1683	8.71990	-10.594	24.9302	0.4171
Control	Palmaria LL	6.3516	9.20470	-12.398	25.1010	0.4951
Palmaria LL	Ulva LL	0.8167	9.77696	-19.098	20.7317	0.9339

Timepoint 2

Ordered Differences Report

Level	- Level	Difference	Std Err Dif	Lower CL	Upper CL	p-Value
Ulva 14C	Ulva LL	603.2420	41.63454	518.328	688.1562	<.0001*
Ulva 14C	Control	602.6840	37.52887	526.143	679.2246	<.0001*
Ulva 14C	Palmaria LL	565.4360	41.63454	480.522	650.3502	<.0001*
Ulva 14C	Palmaria 9C	505.8280	41.63454	420.914	590.7422	<.0001*
Ulva 9C	Ulva LL	458.0880	41.63454	373.174	543.0022	<.0001*
Ulva 9C	Control	457.5300	37.52887	380.989	534.0706	<.0001*
Ulva 9C	Palmaria LL	420.2820	41.63454	335.368	505.1962	<.0001*
Ulva 9C	Palmaria 9C	360.6740	41.63454	275.760	445.5882	<.0001*
Palmaria 14C	Ulva LL	310.6140	41.63454	225.700	395.5282	<.0001*
Palmaria 14C	Control	310.0560	37.52887	233.515	386.5966	<.0001*
Ulva 14C	Palmaria 14C	292.6280	41.63454	207.714	377.5422	<.0001*
Palmaria 14C	Palmaria LL	272.8080	41.63454	187.894	357.7222	<.0001*
Palmaria 14C	Palmaria 9C	213.2000	41.63454	128.286	298.1142	<.0001*
Ulva 9C	Palmaria 14C	147.4740	41.63454	62.560	232.3882	0.0013*
Ulva 14C	Ulva 9C	145.1540	41.63454	60.240	230.0682	0.0015*
Palmaria 9C	Ulva LL	97.4140	41.63454	12.500	182.3282	0.0259*
Palmaria 9C	Control	96.8560	37.52887	20.315	173.3966	0.0148*
Palmaria 9C	Palmaria LL	59.6080	41.63454	-25.306	144.5222	0.1622
Palmaria LL	Ulva LL	37.8060	41.63454	-47.108	122.7202	0.3709
Palmaria LL	Control	37.2480	37.52887	-39.293	113.7886	0.3286
Control	Ulva LL	0.5580	37.52887	-75.983	77.0986	0.9882

Timepoint 4

As the times at which these three experimental conditions were sampled did not align perfectly, we compared the closest possible times. As such, Timepoint 1 refers to a comparison of the 1hr point in the 9C experiment, the 1hr point in the 14C experiment, and the 0.75 hr in the low light condition, the same follows for timepoints 2, 3, and 4.

U. fenestra						*P. palmata*						*Controls*				
$CHBr_3$ Time (hrs)	*$CHBr_3$ Avg.	$CHBr_3$ Std. Dev	H_2O_2 Time (hrs)	$^{x}H_2O_2$ Avg.	H_2O_2 Std. Dev	$CHBr_3$ Time (hrs)	*$CHBr_3$ Avg.	$CHBr_3$ Std. Dev.	H_2O_2 Time (hrs)	$^{x}H_2O_2$ Avg.	H_2O_2 Std. Dev	Time (hrs)	$^{t}CHBr_3$ Avg.	$CHBr_3$ Std. Dev.	$^{a}H_2O_2$ Avg.	H_2O_2 Std. Dev
1	244.87	5.54	0.7	44.21	22.34	2	19.28	0.01	1.7	3.47	3.13	1	39.755	1.18	47.2	8.43
2.17	328.72	23.13	1.87	182.18	155.20	4.5	19.22	10.52	3.7	109.73	54.45	2.17				
4.17	811.27	98.04	3.87	1191.30	572.84	5.75	31.10	5.05	5.45	813.20	134.65	4.17				
7.83	674.18	143.18	7.53	1452.15	549.10	7.0	129.84	27.88	6.7	1447.94	315.53	7.83	44.55	4.32	45.9	13.12
9.17	987.94	148.56	8.87	1645.74	446.93	9.0	64.98	2.67	8.7	1614.54	304.71	9.17				
11.17	1067.30	156.09	10.87	549.25	159.29	10.5	193.52	49.67	10.2	588.62	770.87	11.17				

Table S7. Data for incubations of *U. fenestra* and *P. palmata* incubations at 9˚C and 130 umol m^{-2} s^{-1} light (corresponding to Figure 5). *Average $CHBr_3$ (ng g^{-1} FW) over three biological replicates; ^{x} Average H_2O_2 (nM g^{-1} FW).

Standard Condition					Heat					*Grazing*					Controls					
Hrs	*U. fenestra* H_2O_2 (nM g^{-1} FW)		*P. palmata* H_2O_2 (nM g^{-1} FW)		Hrs	*U. fenestra* H_2O_2 (nM g^{-1} FW)		*P. palmata* H_2O_2 (nM g^{-1} FW)		Hrs	*U. fenestra* H_2O_2 (nM g^{-1} FW)		*P. palmata* H_2O_2 (nM g^{-1} FW)		Heat (nM)		Grazing (nM)		Standard (nM)	
	Avg.	Std. Dev.	Avg.	Std. Dev.		Avg.	Std. Dev.	Avg.	Std. Dev.		Avg.	Std. Dev.	Avg.	Std. Dev.	Avg	Std. Dev	Avg	Std. Dev	Avg	Std. Dev
0	54.30	11.32	48.21	13.92	0	59.20	9.43	48.53	3.28	0	43.78	5.09	42.68	8.49	64.5	8.73	70.8	24.5	68.3	4.58
1	102.34	19.85	60.20	20.50	1	2583.79	35.98	3200.28	28.97	1	5048.07	577.98	55.78	19.41	72.3	13.4	75.3	12.4	78.2	10.20
2.17	205.40	21.34	108.64	23.40	2	2904.71	288.15	3800.91	130.29	2	5259.37	1093.43	110.97	22.43	68.5	18.4	68.2	11.6	66.4	8.52
4.17	363.80	54.46	171.42	38.94	6	4705.28	115.00	4400.15	98.51	6	5249.35	183.45	178.23	17.54	66.3	10.6	60.5	9.7	71.8	12.58
7.83	644.20	71.23	351.68	84.30	9	5644.59	128.88	5100.26	120.38	9	5899.39	699.04	285.65	34.53	70.9	12.1	71.2	14.5	69.8	2.13
9.17	543.40	71.88	283.75	96.07																

Table S8. H_2O_2 (nM g^{-1} FW) in incubation waters of *U. fenestra and P. palmata* under standard conditions (9˚C and 100 umol m^{-2} s^{-1} light), under acute heat stress at 20˚C, and in the presence of grazers (*Littorina littorea)*. Data corresponds to Figure 3. A one-way analysis of variance (ANOVA) of treatment type showed no significant difference between control seawaters (P = 0.7851). Bivariate fits of each control condition (Heat, Grazing, and Standard) revealed no significant trends with time (Grazing R^2 = 0.037831, Heat R^2 = 0.01822, and Standard Condition R^2 = 0.003864).

Ordered Differences Report — TP 1 (0hrs)

Level	- Level	Difference	Std Err Dif	Lower CL	Upper CL	p-Value
Control	Palm Grazing	25.72000	6.437579	10.4975	40.94246	0.0052*
Control	Ulva Grazing	24.61000	6.437579	9.3875	39.83246	0.0065*
Control	Palm Std	20.19000	6.437579	4.9675	35.41246	0.0165*
Control	Palm Heat	19.87000	6.437579	4.6475	35.09246	0.0177*
Ulva Heat	Palm Grazing	16.52000	6.437579	1.2975	31.74246	0.0372*
Ulva Heat	Ulva Grazing	15.41000	6.437579	0.1875	30.63246	0.0479*
Control	Ulva Std	14.10500	6.437579	-1.1175	29.32746	0.0646
Ulva Std	Palm Grazing	11.61500	6.437579	-3.6075	26.83746	0.1142
Ulva Heat	Palm Std	10.99000	6.437579	-4.2325	26.21246	0.1316
Ulva Heat	Palm Heat	10.67000	6.437579	-4.5525	25.89246	0.1414
Ulva Std	Ulva Grazing	10.50500	6.437579	-4.7175	25.72746	0.1467
Control	Ulva Heat	9.20000	6.437579	-6.0225	24.42246	0.1960
Ulva Std	Palm Std	6.08500	6.437579	-9.1375	21.30746	0.3760
Palm Heat	Palm Grazing	5.85000	6.437579	-9.3725	21.07246	0.3937
Ulva Std	Palm Heat	5.76500	6.437579	-9.4575	20.98746	0.4003
Palm Std	Palm Grazing	5.53000	6.437579	-9.6925	20.75246	0.4188
Ulva Heat	Ulva Std	4.90500	6.437579	-10.3175	20.12746	0.4710
Palm Heat	Ulva Grazing	4.74000	6.437579	-10.4825	19.96246	0.4855
Palm Std	Ulva Grazing	4.42000	6.437579	-10.8025	19.64246	0.5144
Ulva Grazing	Palm Grazing	1.11000	6.437579	-14.1125	16.33246	0.8680
Palm Heat	Palm Std	0.32000	6.437579	-14.9025	15.54246	0.9617

TP 1 (0hrs)

Ordered Differences Report — TP 2 (1hr)

Level	- Level	Difference	Std Err Dif	Lower CL	Upper CL	p-Value
Ulva Grazing	Palm Grazing	4992.290	155.2525	4625.18	5359.404	<.0001*
Ulva Grazing	Palm Std	4987.870	155.2525	4620.76	5354.984	<.0001*
Ulva Grazing	Control	4979.670	155.2525	4612.56	5346.784	<.0001*
Ulva Grazing	Ulva Std	4945.730	155.2525	4578.62	5312.844	<.0001*
Palm Heat	Palm Grazing	3144.500	155.2525	2777.39	3511.614	<.0001*
Palm Heat	Palm Std	3140.080	155.2525	2772.97	3507.194	<.0001*
Palm Heat	Control	3131.880	155.2525	2764.77	3498.994	<.0001*
Palm Heat	Ulva Std	3097.940	155.2525	2730.83	3465.054	<.0001*
Ulva Heat	Palm Grazing	2798.010	155.2525	2430.90	3165.124	<.0001*
Ulva Heat	Palm Std	2793.590	155.2525	2426.48	3160.704	<.0001*
Ulva Heat	Control	2785.390	155.2525	2418.28	3152.504	<.0001*
Ulva Heat	Ulva Std	2751.450	155.2525	2384.34	3118.564	<.0001*
Ulva Grazing	Ulva Heat	2194.280	155.2525	1827.17	2561.394	<.0001*
Ulva Grazing	Palm Heat	1847.790	155.2525	1480.68	2214.904	<.0001*
Palm Heat	Ulva Heat	346.490	155.2525	-20.62	713.604	0.0608
Ulva Std	Palm Grazing	46.560	155.2525	-320.55	413.674	0.7730
Ulva Std	Palm Std	42.140	155.2525	-324.97	409.254	0.7939
Ulva Std	Control	33.940	155.2525	-333.17	401.054	0.8332
Control	Palm Grazing	12.620	155.2525	-354.49	379.734	0.9375
Control	Palm Std	8.200	155.2525	-358.91	375.314	0.9594
Palm Std	Palm Grazing	4.420	155.2525	-362.69	371.534	0.9781

TP 2 (1hr)

Ordered Differences Report — TP 3 (2hr)

Level	- Level	Difference	Std Err Dif	Lower CL	Upper CL	p-Value
Ulva Grazing	Control	5190.970	304.3919	4471.20	5910.742	<.0001*
Ulva Grazing	Palm Std	5150.730	304.3919	4430.96	5870.502	<.0001*
Ulva Grazing	Palm Grazing	5148.400	304.3919	4428.63	5868.172	<.0001*
Ulva Grazing	Ulva Std	5053.970	304.3919	4334.20	5773.742	<.0001*
Palm Heat	Control	3732.510	304.3919	3012.74	4452.282	<.0001*
Palm Heat	Palm Std	3692.270	304.3919	2972.50	4412.042	<.0001*
Palm Heat	Palm Grazing	3689.940	304.3919	2970.17	4409.712	<.0001*
Palm Heat	Ulva Std	3595.510	304.3919	2875.74	4315.282	<.0001*
Ulva Heat	Control	2836.310	304.3919	2116.54	3556.082	<.0001*
Ulva Heat	Palm Std	2796.070	304.3919	2076.30	3515.842	<.0001*
Ulva Heat	Palm Grazing	2793.740	304.3919	2073.97	3513.512	<.0001*
Ulva Heat	Ulva Std	2699.310	304.3919	1979.54	3419.082	<.0001*
Ulva Grazing	Ulva Heat	2354.660	304.3919	1634.89	3074.432	0.0001*
Ulva Grazing	Palm Heat	1458.460	304.3919	738.69	2178.232	0.0020*
Palm Heat	Ulva Heat	896.200	304.3919	176.43	1615.972	0.0216*
Ulva Std	Control	137.000	304.3919	-582.77	856.772	0.6663
Ulva Std	Palm Std	96.760	304.3919	-623.01	816.532	0.7598
Ulva Std	Palm Grazing	94.430	304.3919	-625.34	814.202	0.7654
Palm Grazing	Control	42.570	304.3919	-677.20	762.342	0.8927
Palm Std	Control	40.240	304.3919	-679.53	760.012	0.8985
Palm Grazing	Palm Std	2.330	304.3919	-717.44	722.102	0.9941

TP 3 (2hr)

Ordered Differences Report — TP 4 (6hrs)

Level	- Level	Difference	Std Err Dif	Lower CL	Upper CL	p-Value
Ulva Grazing	Control	5180.950	66.24355	5024.31	5337.591	<.0001*
Ulva Grazing	Palm Grazing	5071.120	66.24355	4914.48	5227.761	<.0001*
Ulva Grazing	Palm Std	4999.140	66.24355	4842.50	5155.781	<.0001*
Ulva Grazing	Ulva Std	4749.545	66.24355	4592.90	4906.186	<.0001*
Ulva Heat	Control	4636.880	66.24355	4480.24	4793.521	<.0001*
Ulva Heat	Palm Grazing	4527.050	66.24355	4370.41	4683.691	<.0001*
Ulva Heat	Palm Std	4455.070	66.24355	4298.43	4611.711	<.0001*
Palm Heat	Control	4331.750	66.24355	4175.11	4488.391	<.0001*
Palm Heat	Palm Grazing	4221.920	66.24355	4065.28	4378.561	<.0001*
Ulva Heat	Ulva Std	4205.475	66.24355	4048.83	4362.116	<.0001*
Palm Heat	Palm Std	4149.940	66.24355	3993.30	4306.581	<.0001*
Palm Heat	Ulva Std	3900.345	66.24355	3743.70	4056.986	<.0001*
Ulva Grazing	Palm Heat	849.200	66.24355	692.56	1005.841	<.0001*
Ulva Grazing	Ulva Heat	544.070	66.24355	387.43	700.711	<.0001*
Ulva Std	Control	431.405	66.24355	274.76	588.046	0.0003*
Ulva Std	Palm Grazing	321.575	66.24355	164.93	478.216	0.0018*
Ulva Heat	Palm Heat	305.130	66.24355	148.49	461.771	0.0025*
Ulva Std	Palm Std	249.595	66.24355	92.95	406.236	0.0070*
Palm Std	Control	181.810	66.24355	25.17	338.451	0.0287*
Palm Grazing	Control	109.830	66.24355	-46.81	266.471	0.1413
Palm Std	Palm Grazing	71.980	66.24355	-84.66	228.621	0.3132

TP 4 (6hrs)

Ordered Differences Report — TP 5 (9hrs)

Level	- Level	Difference	Std Err Dif	Lower CL	Upper CL	p-Value
Ulva Grazing	Control	5830.990	195.3148	5369.14	6292.836	<.0001*
Ulva Grazing	Palm Std	5615.640	195.3148	5153.79	6077.486	<.0001*
Ulva Grazing	Palm Grazing	5613.740	195.3148	5151.89	6075.586	<.0001*
Ulva Heat	Control	5576.190	195.3148	5114.34	6038.036	<.0001*
Ulva Grazing	Ulva Std	5360.990	195.3148	4899.14	5822.836	<.0001*
Ulva Heat	Palm Std	5360.840	195.3148	4898.99	5822.686	<.0001*
Ulva Heat	Palm Grazing	5358.940	195.3148	4897.09	5820.786	<.0001*
Ulva Heat	Ulva Std	5106.190	195.3148	4644.34	5568.036	<.0001*
Palm Heat	Control	5031.860	195.3148	4570.01	5493.706	<.0001*
Palm Heat	Palm Std	4816.510	195.3148	4354.66	5278.356	<.0001*
Palm Heat	Palm Grazing	4814.610	195.3148	4352.76	5276.456	<.0001*
Palm Heat	Ulva Std	4561.860	195.3148	4100.01	5023.706	<.0001*
Ulva Grazing	Palm Heat	799.130	195.3148	337.28	1260.976	0.0046*
Ulva Heat	Palm Heat	544.330	195.3148	82.48	1006.176	0.0270*
Ulva Std	Control	470.000	195.3148	8.15	931.846	0.0470*
Ulva Grazing	Ulva Heat	254.800	195.3148	-207.05	716.646	0.2333
Ulva Std	Palm Std	254.650	195.3148	-207.20	716.496	0.2335
Ulva Std	Palm Grazing	252.750	195.3148	-209.10	714.596	0.2367
Palm Grazing	Control	217.250	195.3148	-244.60	679.096	0.3027
Palm Std	Control	215.350	195.3148	-246.50	677.196	0.3067
Palm Grazing	Palm Std	1.900	195.3148	-459.95	463.746	0.9925

TP 5 (9hrs)

Table S9. Results of Student T-tests comparing the means between incubations of *U. fenestrata* and *P. palmata* under heat stress at 20°C, and grazing stress (3 snails of *Littorina littorea)* at 9°C, with standard growth at 9°C, and controls just containing seawater. All incubations were done under 100 µmol m^{-2} s^{-1} light. Data used for these analyses can be found in Table S8.

0 hrs				2 hrs				4 hrs				*Control*	
U. fenestra H_2O_2 (nM g^{-1} FW)		*P. palmata* H_2O_2 (nM g^{-1} FW)		*U. fenestra* H_2O_2 (nM g^{-1} FW)		*P. palmata* H_2O_2 (nM g^{-1} FW)		*U. fenestra* H_2O_2 (nM g^{-1} FW)		*P. palmata* H_2O_2 (nM g^{-1} FW)			
Average	Std. Dev.	Average	Std. Dev.	Average	Std. Dev.	Average	Std. Dev.	Average	Std. Dev.	Average	Std. Dev.	Average	Std. Dev.
57.94	12.28	62.40	5.81	379.33	90.39	401.47	55.38	778.12	110	540.84	98.04	44.7	1.8

Table S10. H_2O_2 in incubation waters within 2 minutes after re-hydration of individuals of *U. fenestra and P. palmata* that had been desiccated for 0 hrs, 2 hrs, and 4 hrs respectively. Data corresponds to Figure 4.

Time (hrs)	Ulva $CHBr_3$ (ng g^{-1} FW): Standard Conditions		Ulva $CHBr_3$ (ng g^{-1} FW): Heat Experiment		Ulva $CHBr_3$ (ng g^{-1} FW): Grazing Experiment		Palmaria $CHBr_3$ (ng g^{-1} FW): Standard Conditions		Palmaria $CHBr_3$ (ng g^{-1} FW): Heat Experiment		Palmaria $CHBr_3$ (ng g^{-1} FW): Grazing Experiment	
	Average	Std. Dev.	Average	Std. Dev.	Average	Std. Dev.	Average	Std. Dev.	Average	Std. Dev.	Average	Std. Dev.
1.3	328.21	23.52	4508.81	306.24	3892.19	794.04	30.75	5.83	43.31	10.01	28.75	9.81
7.3	674.49	243.80	5029.49	826.31	2815.43	494.38	57.53	18.82	55.89	14.84	60.12	21.56

Table S11. Bromoform concentrations (ng g^{-1} FW) in seawater incubations of *U. fenestra and P. palmata* under standard conditions (9˚C and 100 umol m^{-2} s^{-1} light), in an acute heat stress experiment (20˚C and 100 umol m^{-2} s^{-1} light), and under grazing pressure by snails (*Littorina littorea*, 100 umol m^{-2} s^{-1} light). Data corresponds to Figure 6.

Experimental Condition	Difference in H_2O_2 (%) *U. fenestra*	Difference in H_2O_2 (%) P. *palmata*
Heat	1458	7783
Grazers	2722	N/A
High light	50	38
14˚C	-0.87	75
Low Light	-78	-59

Table S12. Difference in H_2O_2 (%) after 2 hours of incubation relative to standard conditions (9˚C under 100 umol m^{-2} s^{-1} light). **Heat**: 20˚C, 100 umol m^{-2} s^{-1} light; **Grazers:** presence of snails (*Littorina littorea)*, 100 umol m^{-2} s^{-1} light; **High light**: 9˚C; 130 umol m^{-2} s^{-1} light, **14˚C**: 14˚C, and 100 umol m^{-2} s^{-1} light; and **Low light**: 9˚C, and 30 umol m^{-2} s^{-1} light. Data corresponds to Figure 7.
